# Five-factor model personality traits, exclusive breastfeeding, and self-efficacy: a mediational analysis

**DOI:** 10.1186/s12884-024-06494-z

**Published:** 2024-04-16

**Authors:** Parvin Yadollahi, Fatemeh Padashian, Marziyeh Doostfatemeh

**Affiliations:** 1 Maternal-fetal Medicine Research Center, Department of Midwifery, School of Nursing and Midwifery, Shiraz University of Medical Sciences, Shiraz, Iran; 2 Department of Midwifery, Behbahan Branch, Islamic Azad University, Behbahan, Iran; 3 Department of Biostatistics, Department of Biostatistics, School of Medicine, Shiraz University of Medical Sciences, Shiraz, Iran

**Keywords:** Breastfeeding, Self-efficacy, Personality traits

## Abstract

**Background:**

Despite the World Health Organization’s (WHO) emphasis on exclusive breastfeeding, the documents show a declining trend worldwide. Studies assert that the mother’s personality traits appear to have an impact on this issue. This study aimed to investigate the potential influence of personality traits on exclusive breastfeeding, which might be channeled by self-efficacy as a mediator variable.

**Methods:**

Data were analyzed from the cross-sectional study. The exclusive breastfeeding scale, the breastfeeding self-efficacy questionnaire, and the Five-Factor Model questionnaire (as follows: neuroticism, extraversion, openness experience, agreeableness, and conscientiousness) were completed by120 Iranian volunteer mothers with an infant aged 6–12 months referred to health centers in Shiraz (a major city in southern Iran) between May to December 2019. The structural equation modeling (SEM) approach was used to obtain the direct and indirect effects of personality traits and self-efficacy on exclusive breastfeeding.

**Results:**

The study showed the significant direct effect of some personality traits (agreeableness, extraversion, and consciousness) and self-efficacy on exclusive breastfeeding. The indirect effect of extraversion on exclusive breastfeeding through self-efficacy was also obtained from the result of SEM analysis. The model fit the data satisfactorily, according to the fit indices criteria extracted from the mediational analysis.

**Conclusions:**

Self-efficacy appears to be a significant predictor of exclusive breastfeeding. Therefore, exclusive breastfeeding could be enhanced by safe education in pregnancy, reinforcing the self-efficacy of pregnant women and considering their personality traits.

## Background

Exclusive breastfeeding is a cornerstone of child survival and child health because it provides essential, irreplaceable nutrition for a child’s growth and development. Therefore, the World Health Organization (WHO) recommends exclusive breastfeeding (EBF) for the first 6 months after birth [1]. Exclusive breastfeeding is defined as receiving breast milk as the sole source of infant nutrition and only using drops and syrups such as vitamins, minerals, and medicines, if necessary [2]. Many studies have shown the profound health benefits for the mother and child of exclusive breastfeeding. They indicated that the risk of breast and ovarian cancer, birth spacing, and type II diabetes would be reduced for the mothers. Moreover, they confirmed that obesity, type I and II diabetes, gastroenteritis, severe lower respiratory tract infections, atopic dermatitis, asthma, and high blood pressure are less common for such children [3–5]. Furthermore, some studies [6–8], support the benefits of exclusive or partial breastfeeding on children’s cognitive, behavioral, and motor development.

According to the current United Nations International Children’s Emergency Fund (UNICEF**)**, only 43% of infants aged 0 to 6 months are exclusively breastfed [9]. This rate has been reported at 37% in low- and middle-income and 20% in high-income countries [10]. According to recent findings in the UK, while a significant majority of mothers initially begin breastfeeding their newborns(81%), there is a gradual decline in breastfeeding rates, with almost half discontinuing breastfeeding after six weeks (48%) and only 25% continuing to do so at the end of six months after delivery [2, 11, 12]. In Iran, the result of a national study reported a rate of 53.1% for exclusive breastfeeding [13].

Some researchers believe the psychosocial and cognitive factors associated with the start and continuance of breastfeeding. They revealed that mothers’ characteristics such as personality traits, self-esteem, self-efficacy, and emotional stability could influence breastfeeding [14, 15]. Considering the relationship between health behaviors and personality traits and the difference between people with these characteristics, it seems that initiation of breastfeeding and the desire to continue it is an individual skill that is related to the mother’s personality traits [16, 17]. According to the big five factors theory proposed by McCrae and Costa, the personality trait consists of neuroticism (people who are typically neurotic, nervous, insecure, fearful, and anxious), extraversion (people who are energetic, ambitious, talkative, optimistic, confident, outgoing, and reward-seeking), openness to experience (people who are curious, original, intellectual, imaginative, artistic, creative, innovative, and flexible), agreeableness (people who are helpful, good-natured, courteous, cooperative, sympathetic, trusting, and forgiving.), and conscientiousness (be careful, responsible and dependable, organized, efficient, hard-working, and achievement-oriented) [18]. Based on these, all dimensions of these personality traits remain stable and people’s personality profiles do not considerably change with experience of delivery pain and breastfeeding [19, 20].

There are a few studies investigating the association between the mother’s personality traits and breastfeeding. Wagner et al. showed that highly extroverted mothers were more inclined to initiate and continue breastfeeding [17]. Brown and Keller et al. and Di Mattei et al. revealed that extraversion, openness to experience, and agreeableness were the most related personality traits that could encourage the initiation and continuance of breastfeeding [11, 21].

According to the global breastfeeding targets for 2025 (increase the rate of exclusive breastfeeding in the first 6 months up to at least 50%), and while the relationship between the personality trait and breastfeeding is well established, researchers have suggested that this is not a direct relationship and more sophisticated methods and analyses should be conducted to truly understand the effect of the personality trait on breastfeeding and its underlying process.

Bandura’s social cognitive theory demonstrated that self-efficacy is the belief in one’s ability to organize and accomplish tasks required to manage prospective situations and it is reached primarily through the personal experience of dominating difficulties. Breastfeeding self-efficacy refers to a woman’s confidence in her ability to breastfeed her infant. The role of breastfeeding self-efficacy towards achieving and sustaining both breastfeeding and EBF has been established in many studies [22, 23]. Findings of one systematic review focused on affecting self-efficacy in successful breastfeeding. They showed attitudes, subjective norms, and self-efficacy influence intentions to breastfeed. Also, they documented that breastfeeding self-efficacy is higher among mothers with positive breastfeeding experiences [24].

To the best of our knowledge, there has been no previous research to express the relationship between breastfeeding and some psychological factors in the form of a single structural model. Therefore, due to people’s differences in personality traits, the present study aims to investigate the potential influence of personality traits on exclusive breastfeeding, which might be channeled by self-efficacy as a mediator variable.

## Method

### Study design

The study was designed based on structural equation modeling (SEM) between May to December 2019 in Shiraz (a major city in southern Iran).

### Data and Sample

The target population was comprised of 120 Iranian volunteer mothers with an infant aged 6–12 months (Mean = 9.5, SD = 2.4) who had experienced exclusive breastfeeding in the first 6 months of life. To determine the ideal sample size for the structural equation prediction model, the rule of thumb was followed, which recommends selecting 10 samples for each model parameter [25]. The data were recruited from 6 health centers of Shiraz University of Medical Sciences as the main clusters between May to December 2019. Mothers were considered for inclusion if they met the “exclusive breastfeeding” concept. They were also asked to provide written informed consent before participating in the research, and their anonymity was guaranteed. Afterward, they were provided with a patient information sheet and other details about the study measurements that needed to be completed. The other inclusion criteria involved singleton pregnancy, lack of any breast disorders, normal vaginal delivery, not using any lactation-inducing medications, not having any medical complications, not having been hospitalized for any reason (neither the infant nor the mother), not using any psychiatric medications during the breastfeeding period and term pregnancy. The fully unanswered questionnaires were also excluded.

### Instruments and measures

#### Exclusive breastfeeding scale

The Exclusive Breastfeeding Scale, which was designed and validated previously in Iran [26], was completed by eligible mothers in this study. This is a 23-item generic instrument that consists of four subscales, including attitude (11 items), abstract norms (7 items), perceived behavioral control (4 items), and behavioral intention (1 item). The participants responded to the items on a five-point Likert scale from 1 (strongly disagree) to 5 (strongly agree). The scale ranged from 23 to 115, with higher scores indicating greater intention to exclusively breastfeed.

#### Breastfeeding self-efficacy questionnaire

The Persian version of Breast-feeding Self-Efficacy (BSES-SF**)**, which was previously translated and validated in Iran, is a 14-item- instrument that measures a mother’s confidence inerrability to successfully breastfeed her infant. All questions begin with “I can always **…”.** Each item is rated on a five-point Likert scale from 1 (Never) to 5 (Always) with higher scores reflecting more significant levels of breastfeeding self-efficacy [27].

#### The five-factor model questionnaire

The Five-Factor Model questionnaire is a self-report 21-item instrument that consists of 5 subscales including neuroticism (with 4 items), extraversion (5 items), openness (4 items), agreeableness (4 items), and conscientiousness (4 items). The participants responded to the items on a five-point Likert scale (1 = strongly disagree, 2 = disagree, 3 = no disagree no agree, 4 = agree and 5 = strongly agree). The validity and reliability of the Persian version of the Five-Factor Model questionnaire were assessed and accepted by Khormaei et al. in 2014 [28].

### Statistical analyses

Descriptive analysis included sociodemographic characteristics of mothers and fathers as well as mothers’ pregnancy history. A baseline analysis including an Independent sample T-test and ANOVA was used to examine the association between the sociodemographic characteristics and mothers’ breastfeeding score.

Structural equation modeling (SEM) was used to investigate the mediation of self-efficacy, the degree to which intermediate variables in a putative causal chain transmit the effect of personality traits on breastfeeding. The mediational modeling permitted estimates of the indirect effect of personality traits on breastfeeding via self-efficacy. Analyses estimated the total effect of personality traits on breastfeeding, partitioning this effect into the direct contribution of personality traits on breastfeeding and the indirect effect of this relationship via self-efficacy. Interpretation of the parameter estimates of the SEM model follows the same logic as the regression coefficients, the one-point increase in the response variable is associated with the estimated change in the predictor.

**Fig. 1:**
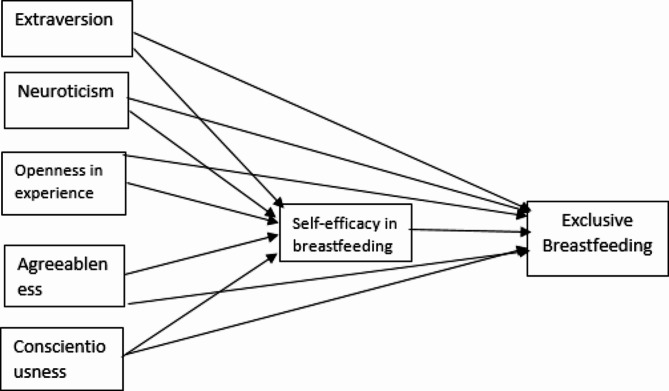
A hypothetical model

Figure 1 depicts the conceptual framework of the hypothesized structural model. To investigate the mediation of self-efficacy, The SEM model was implemented via AMOS software version 24.0, which was based on the maximum likelihood method as the estimation procedure. The strength of fit of the SEM model was investigated based on multiple indices, including the root-mean-square error of approximation (RMSEA), comparative fit index (CFI), Tucker-Lewis index (TLI), and the Normed Fit Index (NFI). The following cut-off values, which were suggested by Hu and Bentler [13], indicate a good fit: RMSEA < 0.06, CFI > 0.95 TLI > 0.95, and NFI > 0.9. A Pearson correlation analysis of the study variables was also performed using the software package SPSS version 25. *P* < 0.05 was considered to indicate a statistically significant correlation.

## Results

Table 1 presents the socio-demographic characteristics and pregnancy history of the study population based on the mothers’ breastfeeding scores. Of the 120 women who were enrolled in the study, the mean score of breastfeeding was 91.1 (SD = 11.9), which indicates that the majority of the mothers had a high grade of breastfeeding. The results showed that the educational level of both fathers and mothers, husband support, gravid, income, and breastfeeding training during pregnancy had no significant effect on breastfeeding scores.

**Table 1:** Sociodemographic characteristics of the study population based on mothers’ BF scores

Variables	N(%) / Mean ± SD	Breastfeed Score(Mean ± SD)	*p*-value
Mother’s age(years)	(31.04 ± 5.10)	(91.1 ± 11.9)	0.851
Monthly income			0.473
Less than a million	15 (12.5%)	(94.9 ± 11.2)	
1–3 million	53 (44.2%)	(91.4 ± 11.9)	
3–6 million	38 (31.7%)	(90.4 ± 12.2)	
Above 6 million	14 (11.7%)	(88.1 ± 11.9)	
Mother’s education			0.888
Academic	58 (48.3%)	(91.1 ± 12.5)	
Non-Academic	62 (51.7%)	(91.3 ± 11.5)	
Father’s education			0.179
Academic	47 (39.2%)	(89.3 ± 12.3)	
Non-Academic	73 (60.8%)	(92.3 ± 11.5)	
Wanted to get pregnant			0.991
yes	98 (81.7%)	(91.1 ± 12.12)	
no	22 (18.3%)	(91.1 ± 11.53)	
Gravid			0.319
once	43 (35.8%)	(92.1 ± 11.4)	
Twice	44 (36.7%)	(89.1 ± 11.5)	
Thrice and more	33 (27.5%)	(93.1 ± 11.3)	
BF training in pregnancy			0.776
yes	107 (89.2%)	(91.2 ± 11.9)	
no	13 (10.8%)	(90.1 ± 11.8)	
Father support in BF			0.1
yes	110 (91.7%)	(90.5 ± 12.1)	
no	10 (8.3%)	(98.7 ± 8.1)	
Mother’s smoking			0.98
yes	4 (97%)	(91.0 ± 15.3)	
no	116 (3%)	(91.1 ± 11.1)	
Mother’s job			0.24
housewife	106 (88.3%)	(91.5 ± 11.7)	
employee	14 (11.7% )	(87.6 ± 13.5)	

BF: Breastfeed

Presented in Table 2 are the results of the correlation analysis of the personality trait subscales with self-efficacy and breastfeeding. A small-to-moderate correlation was found between most of the personality trait subscales and breastfeeding. The highest positive correlation was found for the extraversion component (*r* = 0.36). The openness to experience subscale showed almost no correlation with breastfeeding. Amongst all the personality trait subscales, the extraversion component showed the highest correlation (positive) with self-efficacy, as well (*r* = 0.20). Almost no correlation was found in the rest of the personality subscales.

**Table 2:** Correlation analysis of the study variable

variables	Mean	SD	1	2	3	4	5	6
1. Neuroticism	12.13	2.68	-					
2.Extraversion	13.66	2.14	-0.03	-				
3. Conscientiousness	15.13	1.96	-0.22*	-0.01	-			
4. Agreeableness	16.13	2.10	-0.07	0.07	0.18*	-		
5. Openness to experience	13.97	2.27	-0.03	0.34**	0.17	0.23**	-	
6. Self-efficacy	54.77	7.36	-0.10	0.20*	0.02	-0.08	-0.03	-
Exclusive breastfeeding	91.13	11.92	− 0.11	0.36**	0.13	-0.12	0.008	0.33**

*p ≤ 0.05 **p ≤ 0.01

**Fig. 2:**
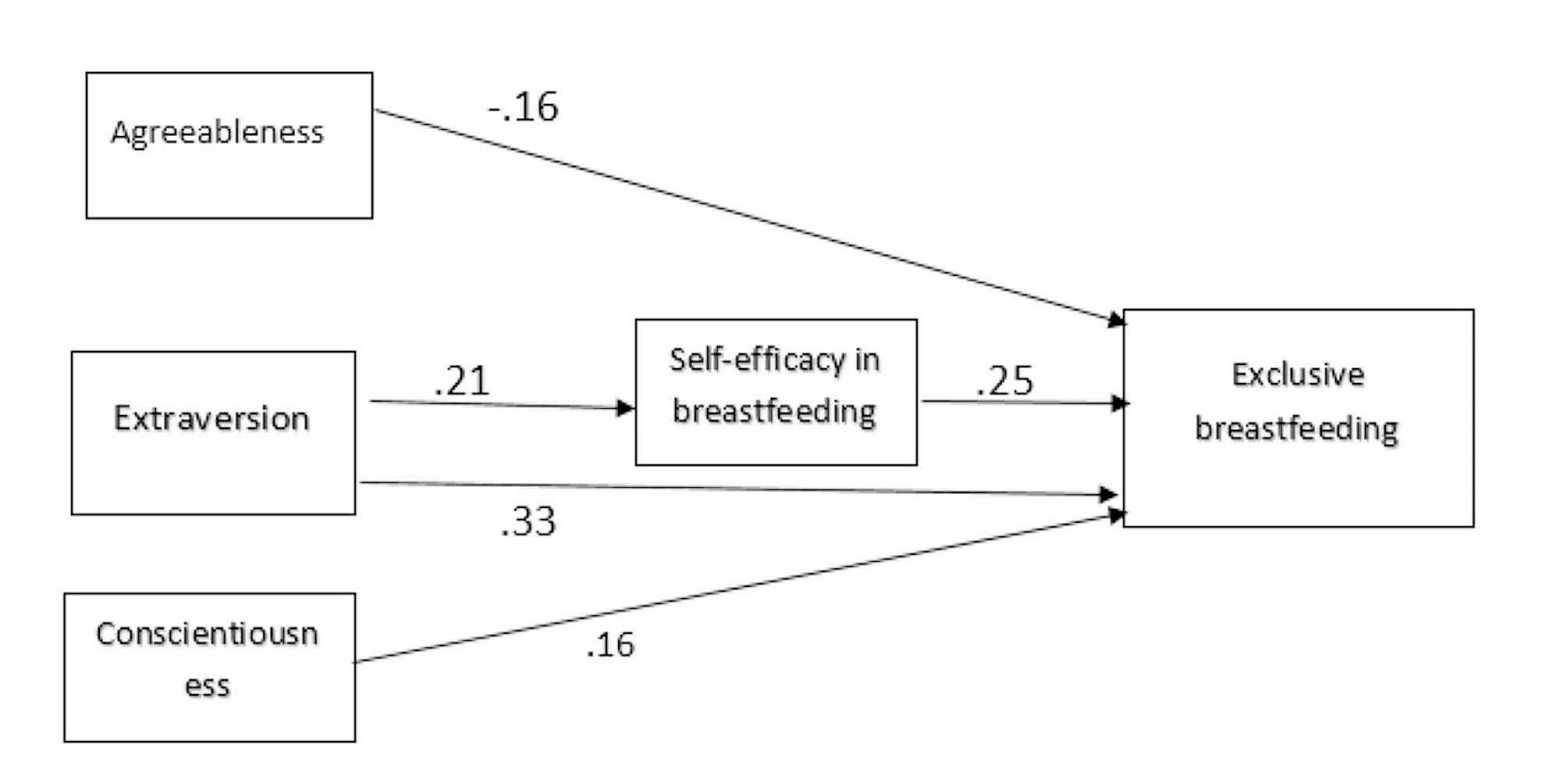
Conceptual model of exclusive breast feeding and personality traits

The results of testing the mediating effect of self-efficacy on the relationship between personality traits subscales and breastfeeding are presented in Table 3. In general, amongst different subscales of five-factor personality traits, only the extraversion subscale affected breastfeeding through self-efficacy. It could be better presented in Fig. 2 which showed a significant direct effect of extraversion on self-efficacy (b = 0.21, *p* = 0.01) as well as self-efficacy on breastfeeding (b = 0.25, *P* = 0.006), indicating an indirect effect of extraversion on exclusive breastfeeding (β = 0.05, *P* = 0.01). The path model also indicates the direct effect of extraversion and conscientiousness on breastfeeding (b = 0.33, *p* < 0.01 and b = 0.16, *p* < 0.05, respectively). Other subscales did not affect exclusive breastfeeding, either directly or through the mediating variable. Model statistics showed that the model adequately fit the data (RMSE = 0.04, CFI = 0.96, TLI = 0.93, and CMIN/DF = 1/23).

**Table 3:** Direct & indirect effects of extraversion on exclusive breastfeeding

variables	path	Direct effect	Significance level	Indirect effect	Significance level	Total effect	Significance level
Extraversion		*0.33*	0.01	0.05	0.01	0.38	0.01
Conscientiousness	Breastfeeding	0.16	0.04	-	NS	0.16	0.04
Agreeableness		-0.16	0.03	-	NS	-0.16	0.03
Extraversion	Self-efficacy	0.21	0.01	-	NS	0.21	0.01
Self-efficacy	Breastfeeding	0.25	0.006	-	NS	0.25	0.006

*p ≤ 0.05 **p ≤ 0.01, NS = non-significant

## Discussion

To the best of the authors’ knowledge, this is the first study that investigates the complex relationship between exclusive breastfeeding and personality traits while mediating self-efficacy via SEM analysis. In this regard, a significant relationship was found between exclusive breastfeeding and personality traits channeled by self-efficacy.

Moreover, the present study found no significant relationship between sociodemographic characteristics and exclusive breastfeeding in mothers. However, other research have shown that various factors can influence a woman’s decision to breastfeed, such as age, education, ethnicity, income, employment status, partner support, and commercial pressure [29]. This issue may be attributed to cultural differences between Iran and other countries. In Iran, exclusive breastfeeding is considered a cultural privilege for women that has been passed down from previous generations. Additionally, most pregnant women in the country receive education on the benefits of exclusive breastfeeding during pregnancy and after delivery at health centers. Taking to gather, There is increasing evidence to support the prominent role of psychosocial factors in predicting breastfeeding intention and duration, including attitudes, knowledge, and beliefs toward infant feeding, compared with alternate biological, demographic, and socioeconomic factors.

The final model results highlighted the indirect effect of the extraversion component on exclusive breastfeeding through self-efficacy. It means that a person’s confidence and belief play an essential role in health-psychological models and lead to successful breastfeeding [12]. Therefore, self-efficacy is a serious determinant of maternal competency for their baby’s breastfeeding immediately and after postpartum [30, 31]. In line with the current study, Economou et al. found that the mother’s confidence in breastfeeding and the newborn’s intention to do so are strong predictors of breastfeeding initiation and continuation [32]. Likewise, Wallenborn et al. described how remarkably workplace support indirectly affected breastfeeding duration through self-efficacy [33]. Brown documented that extroverted mothers continue this action with high confidence and self-efficacy, whereas introverted mothers discontinue breastfeeding due to feeling embarrassed and low confidence [11]. These findings suggest that exclusive breastfeeding, like other healthy behaviors, is greatly influenced by some personality traits, such as extraversion Self-efficacy based on confidence helps with breastfeeding initiation and continuation. Participation in midwife-led breastfeeding support groups increases general self-efficacy and duration of breastfeeding [23].

Our results demonstrated a direct, significant relationship between personality traits such as agreeableness, extraversion, and consciousness with exclusive breastfeeding. Results from a literature review stated the direct effect of the agreeableness, extraversion, and openness experience components on the continuation of breastfeeding [16, 30]. According to Brown and colleagues, certain personality traits such as extraversion, emotional stability, and openness seem to be strongly linked to breastfeeding. However, the research conducted by Keller et al.found no evidence supporting the impact of extraversion on breastfeeding [11, 34]. Wagner also discovered that extraversion impacts breastfeeding continuity beyond the puerperium [17]. Extroverted People tend to be optimistic, assertive, and have a positive outlook on life. They often seek out excitement and new experiences. This positive attitude can lead to a greater acceptance of breastfeeding, which they may view as a way to cope with other challenges and reduce anxiety. Studies have shown that extroverted individuals are more likely to exclusively breastfeed their babies for longer periods compared to those who score lower on measures of extraversion [35, 36].

Another trait we confirmed in relationship with exclusive breastfeeding was the conscientiousness component. It included responsibility, orderliness, and dependability for the owner. In line with the current study, Brown found that conscientiousness was significantly related to the belief that breastfeeding is a healthy behavior, which increased mothers’ motivation to breastfeed but is not a guarantee of continued breastfeeding [37]. In contrast with this study that conscientiousness and breastfeeding together improve physical health, Padashian et al. and Keller et al. demonstrated that there is no relationship between breastfeeding and conscientiousness or situational psychological actions [34, 38].

Agreeableness was one of the components that affected exclusive breast feeding. In accordance with the present study, Turner et al. revealed certain correlations between maternal personality traits of openness, experience, and agreeableness with the breastfeeding period [39]. This component is characterized by good-tempered, friendly, and confident individuals. Therefore, it might be effective in encouraging women to accept that they are able to have prolonged breastfeeding [11].

Finally, neuroticism and openness experience were other maternal personality traits that were not confirmed in the present study, but Di Mattei et al. and Sutin et al. demonstrated the influence of these traits on the duration of breastfeeding [21, 38].

### Strength and limitation

As far as we know, this is the first study to investigate the link between personality traits and exclusive breastfeeding in Iranian mothers, using self-efficacy as a mediator in a single structural model. The second strength of the present study is that the results reported by mothers who exclusively breastfeed are more reliable than those reported by caregivers.

This study also has some limitations which should be taken into consideration before interpreting the results. First, this study faced challenges in selecting mothers who are willing and able to exclusively breastfeed for the first 6 months, while also meeting our other exclusion criteria. Although our relatively small sample size might restrict the generalizability of the finding, according to sample size guidelines for our prediction model, this study meets the methodological acceptance [25]. Second, various psychological, cultural, and social factors can influence a woman’s decision to breastfeed, and these can be considered as mediating factors [40]. Although the current study did not find a significant relationship between certain sociodemographic factors and exclusive breastfeeding, it remains essential to conduct further research with a larger sample size to explore the potential impact of more cultural and psychological factors which could provide valuable insights into why mothers may desire exclusive breastfeeding for their babies. The third limitation is the use of self-reported data for exclusive breastfeeding which may introduce bias. Including objective measures, such as observed breastfeeding behaviors could enhance the validity of the findings. As the last, while this study proposes a conceptual framework, the cross-sectional design limits the ability to establish causality. In this regard, longitudinal studies could help elucidate the temporal relationships between personality traits, self-efficacy, and breastfeeding outcomes.

The result of the present study raises interesting questions about the underlying mechanisms by which personality traits influence self-efficacy and breastfeeding behavior, which could be explored in future research.

## Conclusion

This study found that a mother’s personality traits, such as extraversion, consciousness, and agreeableness, directly impact on exclusive breastfeeding and extraversion has an added effect through self-efficacy. These findings highlight the need to focus on building self-efficacy in mothers to improve maternal and child health outcomes and shed light on the critical role of self-efficacy as a fundamental personality trait in predicting mothers’ breastfeeding initiation and duration. This could help healthcare professionals and policymakers develop effective interventions to support breastfeeding among mothers.

Based on the research, healthcare professionals with a strong educational background can encourage expectant mothers to breastfeed, with an emphasis on exclusive breastfeeding. To achieve this objective, it is essential to create a focus group that serves as a platform for discussion and information sharing about exclusive breastfeeding, while also strengthening the skills required to promote this approach, with a focus on personality traits.

## Data Availability

The data that support the findings of this study are available from the corresponding author, upon reasonable request.

## Abbreviations

WHO: Health Organization’s
SEM: Structural equation modeling
EBF: Exclusive breastfeeding
UNICEF: United Nations International Children’s Emergency Fund
BSES-SF: Breast-feeding Self-Efficacy
RMSEA: Root-mean-square error of approximation
CFI: Comparative fit index
TLI: Tucker-Lewis index
NFI: Normed Fit Index
